# Hospitals and Charitable Aid in New Zealand

**Published:** 1915-02-13

**Authors:** 


### HOSPITALS AND CHARITABLE AID IN NEW ZEALAND.*

### A Glance at State Aid in the Colonies.

The hospitals of Australia are, in part or wholly, dependent upon the State Governments for their funds and are more or less under State control, although the institutions of the separate States vary greatly in forms of administration. But the methods of New Zealand differ from those of the Commonwealth in so much that a more comprehensive attempt has been made to deal with the matter of hospitals and charitable aid, and that throughout the Colony there is joint Municipal and State control. For the purposes of main administration there are thirty-six districts each in charge of a Hospital and Charitable Aid Board which is composed of from eight to twenty members, the body including representatives of the various local bodies of the district, the number of the representatives of each body being fixed by Government regulation, thus giving an important city area greater representation than a small town district. As an instance, Wellington, a large area, includes the City of Wellington, seven Borough Councils, three County Councils, and two Town Boards. The hospitals passed under the control of these Boards, elected by the ratepayers, in accordance with an Act which came into force in 1910; but as it must be obvious that the Boards may be composed of persons not possessing necessary knowledge and experience, it is fortunate that they have power to associate with themselves committees or persons to advise on particular points or to administer special institutions. So far the system, as described, is largely municipal as to control. The Government steps in when it is proposed to appoint an important officer, as no such appointment can be made until three weeks after the Inspector-General of Hospitals has been notified of the intention to make the appointment.

### The Subsidy.

The Minister of Finance exercises the power of the purse, and it is only when he is satisfied that a Board has insufficient funds to carry on that subsidies are made under a plan detailed in the Act. The subsidy shall not exceed ten shillings for every pound value of devises or bequests of not over £500 in value, twenty-four shillings for every pound of voluntary contributions, and for every pound levied on contributory local authorities £1 in respect of capital expenditure and an amount dependent upon rateable value per head in respect of other than capital expenditure. The Inspector-General has wide powers in respect of inspection, calling meetings of Boards, and other matters.

The Annual Report of the Inspector-General of Hospitals, who is also the Chief Health Officer, is an imposing volume of 120 pages. A considerable part of the Report and a large appendix deals with the administration of the Public Health Acts, and information in respect of these matters is necessarily sometimes intermingled with particulars of hospital and charitable aid matters; subject to this the remainder of the Report and an appendix of about fifty pages is concerned with hospital and charitable aid and kindred subjects.

The Report shows that £660,464 (an increase of £76,791 over the previous year) was received by Boards, separate institutions, and Government institutions (the latter appear to consist of the Waikato Sanatorium and the St. Helen's Hospital, with an expenditure together of £14,329). The receipts are divided under four headings: Government contributions, £219,520; levies on local authorities, £175,120; voluntary contributions, £49,856; and payments by persons relieved, £84,026.

### Voluntary Gifts.

Comparing these figures with those of the three previous years an annual increase is shown each year, except in respect of voluntary contributions, which fluctuate from year to year as follows:— 1910-11, £35,433; 1911-12, £31,656; 1912-13, £25,930; 1913-14, £49,856. But it should be noted that the last total includes a gift from one man of £15,000. Roughly calculated, the voluntary contributions in the good year 1913-14 are only 7½ per cent. of the total revenue.

Provision for the sick is of course increased from time to time, the latest important addition being the King George V. Coronation Memorial Hospital for Consumptives, opened in North Canterbury in June last. Expenditure on account of hospitals has increased in four years from £262,241 to £411,454. The Report refers to the working of the Nurses' Registration and Midwives Acts, and foreshadows an Act to provide for the registration of masseurs.

From the appendix dealing with Public Health some interesting information may be gleaned. The birth-rate of the Colony has decreased in the year reviewed by 0.34 per cent. to 26.14 per 1,000. The number of male children born during 1913 was 14,433, and of female children 13,502. The total number of births registered has increased from 19,135 in 1887 to 27,935 in 1913, while the birth-rate has fallen in the same period from 32.09 per 1,000 to 26.14. The proportion of births to each marriage has fallen from 4.37 in 1893 to 2.81 in 1913. The illegitimacy rate has kept steadily between 4 and 5 per 1,000 births for ten years, and is lower than that of the Australian States and Tasmania. During the same decade the proportion of deaths of infants under one year to every 1,000 births has fallen from 70.98 to 65.03.

### Inspection of Institutions.

The second appendix, on Hospitals and Charitable Aid, contains the Reports of the visits of the Inspector-General and his deputies to individual institutions; these are very friendly and practical in character, and should greatly strengthen the hands of the Boards in their administration.

* Report on Public Health and Hospitals and Charitable Aid for the Year 1913-14.

As the following amusing extract from a Report shows, the inspectors do not confine their investigations to the state of the buildings. "The system of keeping accounts was discussed. On examination the apparent reduction in the cost per bed of the hospital appearing in last year's Report could not be substantiated, and the chairman frankly admitted that a mistake had been made. Emulation between hospital boards in the efficient and economical management of their institutions is much to be desired, but a great and sudden reduction in the cost per bed which cannot be verified by examination is, to put it mildly, somewhat inartistic." The Report contains a series of tables showing the receipts and expenditure of each individual institution.

